# Global trends in cutaneous malignant melanoma incidence and mortality

**DOI:** 10.1097/CMR.0000000000000959

**Published:** 2024-02-21

**Authors:** Giuseppe De Pinto, Silvia Mignozzi, Carlo La Vecchia, Fabio Levi, Eva Negri, Claudia Santucci

**Affiliations:** a Department of Clinical Sciences and Community Health, University of Milan, Milan, Italy; b Department of Epidemiology and Health Services Research, Centre for Primary Care and Public Health (Unisanté), University of Lausanne, Lausanne, Switzerland; c Department of Medical and Surgical Sciences, University of Bologna, Bologna, Italy

**Keywords:** incidence, melanoma skin cancer, mortality, public awareness, UV radiation skin

## Abstract

Mortality from cutaneous malignant melanoma (CMM) increased in the past, but trends have been favorable in more recent years in many high-income countries. However, incidence has been increasing in several countries. We provided an up-to-date overview of mortality trends from CMM. We analyzed death certification data from the WHO in selected countries worldwide from 1980 to the most recent available calendar years. We also reported incidence data derived from Cancer Incidence in Five Continents from 1990 to 2012. Separate analyses were performed for young adults aged 20–44 and middle-aged adults aged 45–64 years. Mortality from CMM in all age groups showed a favorable pattern in the majority of the countries considered. Mortality trends declined by 40 to 50% in Australia over the last decades, confirming the importance of prevention measures. Considering young adults aged 20–44, Australia, New Zealand and Northern Europe reported the highest death rates for both sexes (>0.90/100 000 in men and >0.60/100 000 in women) while Japan, the Philippines, and Latin America the lowest ones (<0.50/100 000 and <0.35/100 000 in men and women, respectively). Incidence trends were stable or upward in most countries, with higher rates among women. Our study highlights a global reduction of CMM mortality over the last three decades. The increasing awareness of risk factors, mainly related to UV exposure, along with early diagnosis and progress in treatment for advanced disease played pivotal roles in reducing CMM mortality, particularly in Australia.

## Introduction

Cutaneous malignant melanoma (CMM) accounted for 325 000 incident cases and 57 000 deaths worldwide in 2020, according to the Global Cancer Observatory [1]. Although the incidence of CMM tends to increase with age, it is a relatively common cancer in young adults [2]. The highest incidence and mortality are reported in Australia and North American countries and the lowest ones in Asia and Southern America [3]. After substantial rises up to the 1990s in most high-income countries, mortality has leveled off, while incidence rates have remained upward [4]. The key risk factor of CMM is the exposure to ultraviolet (UV) radiation [5].

Updated quantification of incidence and mortality trends, along with their international comparison, is important for increasing awareness of risk factors, assessing health needs, and allocating resources. To that purpose, we provided incidence and mortality trends over the most recent decades in selected major countries worldwide.

## Methods

### Mortality data

We analyzed official death certification data from skin cancer provided by the WHO database [6] from 1980 to 2019, except for France (2017), Norway (2016), and Venezuela (2016). Our selection of countries was based on criteria including population size (for European ones over 5 000 000 inhabitants and other countries worldwide with over 20 000 000), data coverage (≥90%), and data quality (high or medium), as declared by the WHO [7]. Additionally, we included analysis of the European Union (EU).

To define deaths from melanoma (ICD 10th: ‘C43’) and non-melanoma skin cancer (ICD 10^th^: ‘C44’) for our analysis we considered only young adults aged 20–44 and middle-aged adults aged 45–64 years. In these age groups, approximately 90% and 80% of skin cancer deaths are respectively attributed to CMM [8].

Estimates of the resident population, based on official censuses, were obtained from the same WHO database [6]. Using the matrices of certified deaths and resident populations, we calculated age-specific rates for each 5-year age group (from 20–24 to 40–44 years and from 45–49 to 60–64) and calendar periods. Age-standardized mortality rates (ASMRs) per 100 000 were computed using the direct method and the world standard population. ASMRs for the quinquennia 2005–2009 and 2015–2019 were reported, along with the corresponding change in rates between the two periods.

For the 11 most populous countries (i.e. Australia, New Zealand, Japan, the Philippines, Argentina, Brazil, Colombia, Mexico, Venezuela, Canada, and the USA) plus the EU, we applied joinpoint regression model, allowing up to four joinpoints [9]. These models allow us to identify the years characterized by a significant change in mortality rates during the study period, called ‘joinpoints’, when a change in the linear slope (on a log scale) of the temporal trend occurred. To summarize these trends, we estimated the annual percent change (APC) for each identified linear segment, and the weighted average APC over the entire study period.

### Incidence data

We collected information on CMM incidence and the corresponding population data from the Cancer Incidence in Five Continents (CI5plus) database [10]. This is a source of high-quality cancer incidence data provided by national and subnational population-based cancer registries. We considered countries included for mortality that also provided incidence data, for the calendar years 1990–2012. For countries with multiple cancer registries, we aggregated data and restricted analyses to the longest common calendar period between registries to ensure the highest geographic coverage. For each country and sex, we derived annual age-adjusted incidence rates for CMM at 20–44 and 45–64 years. Additionally, we presented the trend in incidence rates using three-year moving averages.

No ethics committee approval was required since we only considered public data. Statistical analyses were performed using the software R version 4.2.0 (R Development Core Team, 2022), SAS version 9.4 (SAS Institute Inc., Cary, NC, USA), and Joinpoint Regression Program version 4.9.1 (Statistical Methodology and Applications Branch, Surveillance Research Program, National Cancer Institute).

## Results

### Mortality

**Fig. 1:**
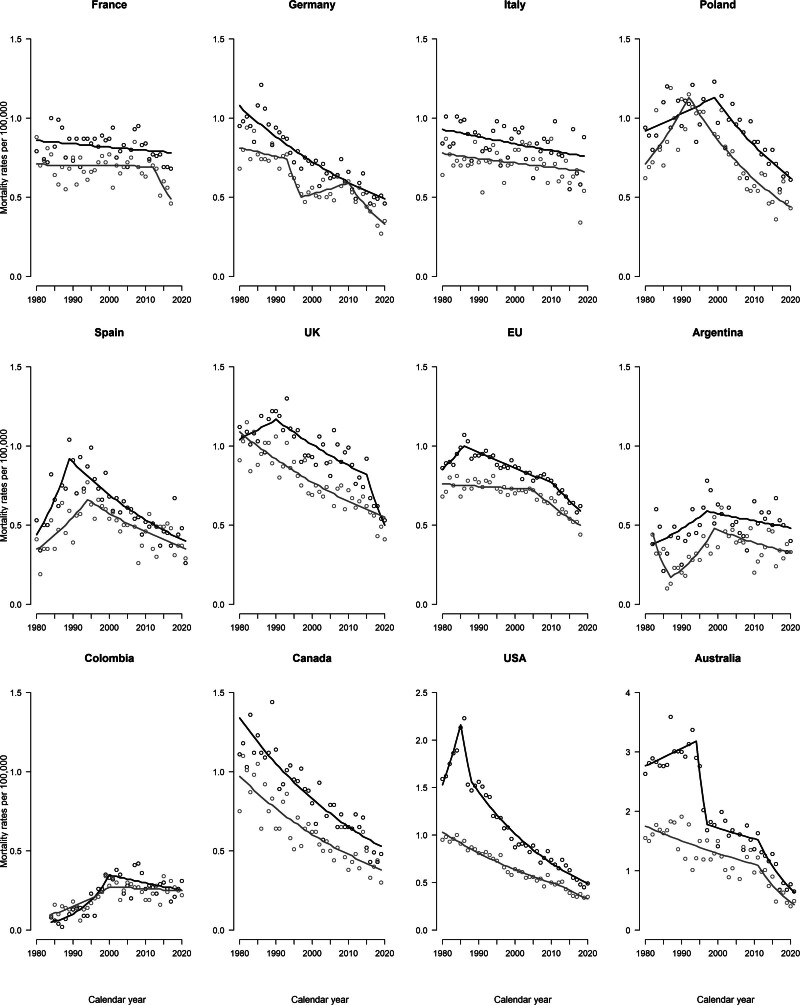
Trends in age-standardized (world population) mortality rates (dots) per 100 000 persons and corresponding joinpoint models (lines) for cutaneous malignant melanoma in selected major countries worldwide, among men (black) and women (gray) aged 20–44.

Table 1 gives the ASMRs from CMM among individuals aged 20–44 years, per 100 000 men and women, in 2005–2009 and 2015–2019, the annual average deaths, and the corresponding change in rates in selected countries worldwide. Figure 1 displays the corresponding trends in ASMRs along with the joinpoint models for the 11 major countries and the EU, for both males and females. Supplementary Table 1a, Supplemental digital content 1, http://links.lww.com/MR/A372 provides detailed results from the joinpoint analyses.

Trends in male mortality from CMM among young adults were favorable in most countries considered. Exceptions were the Czech Republic (+18.2%) and Argentina (+15.9%). For both analyzed quinquennia, the highest male ASMRs were reported in New Zealand, followed by Australia, and selected northern European countries (Belarus, Denmark, Sweden, Slovakia, the Netherlands, Norway, and Hungary), while the lowest rates in Japan and the Philippines, followed by Latin American countries. In 2015–2019, the EU male ASMR was 0.62/100 000 men, −22.5% compared to 2005–2009. The USA and Canada have lower CMM mortality in the young (20–44) and in this age group Australia has mortality not appreciably higher than most European countries. Similarly, trends in female mortality from CMM showed a decline over time in most countries considered (from −2.3% in Spain to −52.9% in New Zealand), except for the Czech Republic (+7.5%), Colombia (+3.7%), and the Philippines (+36.4%). For both quinquennia the highest female rates were recorded in Norway, followed by New Zealand, the Netherlands, and Australia. The lowest female rates, for both quinquennia, were registered in Japan and the Philippines. In the EU, the ASMR was 0.51/100 000 among women during 2015–2019, −23.9% compared to 2005–2009.

**Table 1:** Age-standardized mortality rates from melanoma skin cancers at 20–44 years per 100 000 person-years in 2005–2009 and 2015–2019 (unless indicated in parenthesis), annual average deaths and the corresponding change in rates along with 95% confidence intervals (CI) in selected countries worldwide

	Men					Women				
	Annual average deaths 2005–2009	ASMR 2005–2009	Annual average deaths 2015–2019	ASMR 2015–2019	Percent change 95% CI	Annual average deaths 2005–2009	ASMR 2005–2009	Annual average deaths 2015–2019	ASMR 2015–2019	Percent change 95% CI
Europe										
Austria	14	0.78	10	0.61	−21.8 (−29.8 to −13.8)	13	0.74	8	0.49	−33.8 (−47.4 to −20.2)
Belarus	20	1.06	12	0.68	−35.8 (−58.5 to −13.1)	15	0.78	9	0.44	−43.6 (−75.2 to −12.0)
Belgium	20	0.95	12	0.55	−42.1 (−56.9 to −27.3)	16	0.79	8	0.41	−48.1 (−67.8 to −28.4)
Bulgaria	11	0.71	9	0.59	−16.9 (−23.6 to −10.2)	11	0.74	7	0.58	−21.6 (−30.8 to −12.4)
Czech Republic	12	0.55	15	0.65	18.2 (11.8 to 24.6)	10	0.53	11	0.57	7.5 (4.6 to 10.4)
Denmark	13	1.20	5	0.50	−58.3 (−85.4 to −31.2)	10	0.92	5	0.47	−48.9 (−73.4 to −24.4)
France (2017)	94	0.84	77	0.68	−19.0 (−22.0 to −16.0)	85	0.73	62	0.54	−26.0 (−30.5 to −21.5)
Germany	112	0.64	70	0.50	−21.9 (−24.9 to −18.9)	91	0.56	51	0.38	−32.1 (−37.1 to −27.1)
Greece	15	0.66	9	0.47	−28.8 (−39.4 to −18.2)	10	0.46	8	0.38	−17.4 (−24.7 to −10.1)
Hungary	23	1.23	18	0.90	−26.8 (−34.3 to −19.3)	19	0.99	11	0.59	−40.4 (−54.0 to −26.8)
Italy	98	0.80	76	0.72	−10.0 (−11.4 to −8.6)	84	0.69	61	0.56	−18.8 (−21.7 to −15.9)
Netherlands	50	1.52	23	0.81	−46.7 (−57.0 to −36.4)	42	1.30	20	0.68	−47.7 (−59.2 to −36.2)
Norway (2016)	13	1.41	10	0.94	−33.3 (−50.5 to −16.1)	13	1.42	8	0.81	−43.0 (−66.7 to −19.3)
Poland	64	0.89	52	0.67	−24.7 (−28.8 to −20.6)	44	0.62	38	0.48	−22.6 (−27.1 to −18.1)
Portugal	16	0.74	10	0.53	−28.4 (−39.9 to −16.9)	11	0.53	8	0.42	−20.8 (−30.5 to −11.1)
Romania	34	0.77	28	0.68	−11.7 (−14.4 to −9.0)	27	0.66	21	0.57	−13.6 (−17.1 to −10.1)
Serbia	16	1.18	15	1.11	−5.9 (−7.8 to −4.0)	14	1.01	9	0.70	−30.7 (−42.1 to −19.3)
Slovakia	9	0.79	8	0.67	−15.2 (−22.5 to −7.9)	9	0.83	7	0.56	−32.5 (−48.1 to −16.9)
Spain	56	0.55	46	0.48	−12.7 (−15.0 to −10.4)	42	0.44	38	0.43	−2.3 (−2.8 to −1.8)
Sweden	17	1.03	10	0.57	−44.7 (−61.5 to −27.9)	16	0.97	12	0.66	−32.0 (−43.7 to −20.3)
Switzerland	10	0.66	6	0.39	−40.9 (−59.2 to −22.6)	10	0.65	5	0.32	−50.8 (−75.6 to −26.0)
UK	113	0.96	77	0.67	−30.2 (−34.1 to −26.3)	87	0.72	62	0.54	−25.0 (−28.7 to −21.3)
EU	704	0.80	512	0.62	−22.5 (−23.7 to −21.3)	579	0.67	409	0.51	−23.9 (−25.4 to −22.4)
North America										
Canada	48	0.72	32	0.47	−34.7 (−41.7 to −27.7)	33	0.52	26	0.39	−25.0 (−30.8 to −19.2)
USA	458	0.81	302	0.53	−34.6 (−36.8 to −32.4)	297	0.53	208	0.36	−32.1 (−34.7 to −29.5)
Latin America										
Argentina	30	0.44	42	0.51	15.9 (12.5 to 19.3)	30	0.42	29	0.34	−19.0 (−23.4 to −14.6)
Brazil	166	0.44	160	0.37	−15.9 (−17.5 to −14.3)	119	0.31	124	0.28	−9.7 (−10.8 to −8.6)
Colombia	27	0.34	22	0.24	−29.4 (−36.8 to −22.0)	24	0.27	28	0.28	3.7 (2.8 to 4.6)
Mexico	56	0.28	60	0.26	−7.1 (−8.3 to −5.9)	52	0.24	60	0.24	0.0 (0.0 to 0.0)
Venezuela (2016)	21	0.40	21	0.37	−7.5 (−10.2 to −4.8)	17	0.32	15	0.25	−21.9 (−31.3 to −12.5)
Australasia										
Australia	65	1.56	42	0.91	−41.7 (−49.3 to −34.1)	49	1.21	29	0.63	−47.9 (−58.3 to −37.5)
New Zealand	16	2.07	9	1.09	−47.3 (−72.1 to −22.5)	12	1.40	6	0.66	−52.9 (−86.9 to −18.9)
Japan	26	0.11	22	0.10	−9.1 (−11.5 to −6.7)	30	0.14	23	0.11	−21.4 (−26.8 to −16.0)
Philippines	22	0.14	23	0.12	−14.3 (−18.4 to −10.2)	18	0.11	28	0.15	36.4 (25.4 to 47.4)

ASMR, age-standardized (world population) mortality rate.

**Fig. 2:**
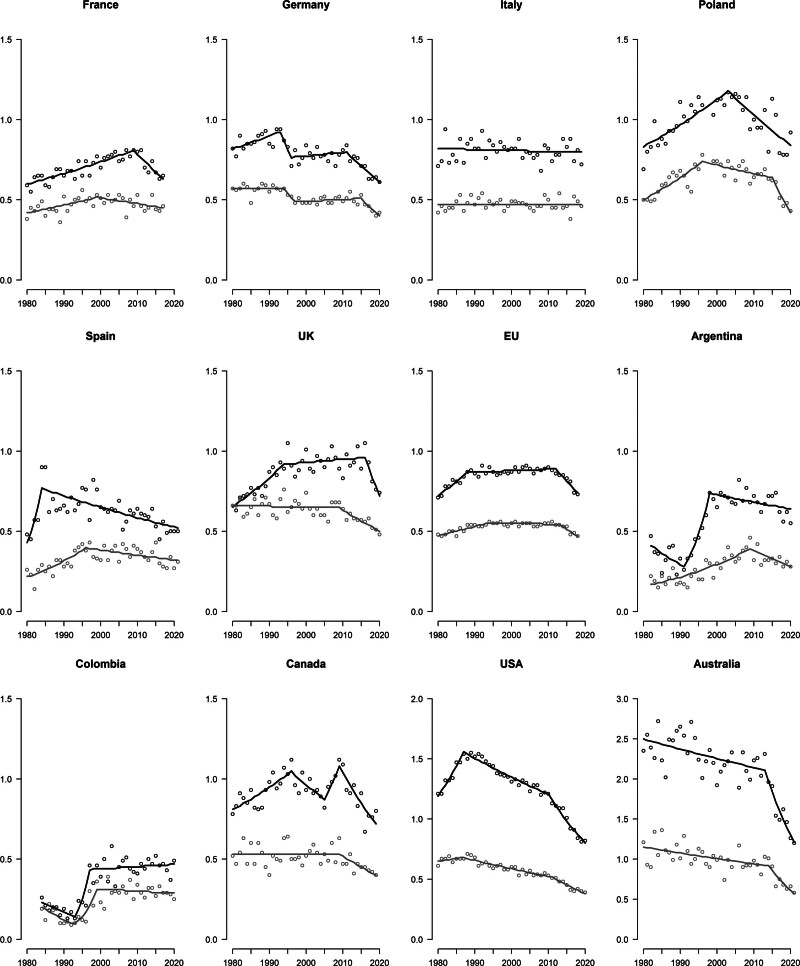
Trends in age-standardized (world population) mortality rates (dots) per 100 000 persons and corresponding joinpoint models (lines) for cutaneous malignant melanoma in selected major countries worldwide, among men (black) and women (gray) aged 45–64.

Table 2, along with Fig. 2, provide the corresponding data for men and women aged 45–64 years. Results from the joinpoint analyses are reported in Supplementary Table 1b, Supplemental digital content 1, http://links.lww.com/MR/A372. The patterns in middle-aged adults were similar to those observed in young adults, with higher rates but consistent trends. Australia, New Zealand, and North Europe reported the highest ASMRs for both sexes while Japan, the Philippines, and Latin America had the lowest ones. Mortality in men declined in almost all analyzed countries, ranging from −0.9% in Greece to −36.8% in Switzerland, except for Belarus (+22.4%), Italy (+3.2%), Romania (+8.4%), Slovakia (+5.6), Venezuela (±36.8%), and the Philippines (+7.5%), which recorded increases between the two time periods. Similarly, female mortality decreased in most countries considered, from −1.6% in Italy to −27.3% in the Czech Republic, except for Bulgaria (+11.3%), Greece (+11.5%), Romania (+11.8%), Sweden (+1.3%), Venezuela (+36.9%), and the Philippines (+7.1%).

**Table 2:** Age-standardized (world population) mortality rates from melanoma skin cancers at 45–64 years per 100 000 person-years in 2005–2009 and 2015–2019 (unless indicated in parenthesis), annual average deaths and the corresponding change in rates along with 95% confidence intervals (CI) in selected countries worldwide

	Men					Women				
	Annual average deaths 2005–2009	ASMR 2005–2009	Annual average deaths 2015–2019	ASMR 2015–2019	Percent change 95% CI	Annual average deaths 2005–2009	ASMR 2005–2009	Annual average deaths 2015–2019	ASMR 2015–2019	Percent change 95% CI
Europe										
Austria	61	5.82	52	4.03	−30.8 (−35.9 to −25.7)	33	3.01	35	2.65	−12.0 (−14.6 to −9.4)
Belarus	43	3.98	60	4.87	22.4 (15.4 to 29.4)	51	3.74	54	3.38	−9.6 (−12.6 to −6.6)
Belgium	66	4.70	59	3.69	−21.5 (−25.1 to −17.9)	46	3.29	46	2.99	−9.1 (−10.9 to −7.3)
Bulgaria	52	5.06	39	3.88	−23.3 (−27.7 to −18.9)	27	2.39	27	2.66	11.3 (8.6 to 14.0)
Czech Republic	83	5.69	63	4.31	−24.3 (−27.9 to −20.7)	55	3.55	38	2.58	−27.3 (−32.4 to −22.2)
Denmark	52	6.60	41	5.21	−21.1 (−25.0 to −17.2)	34	4.44	29	3.77	−15.1 (−18.5 to −11.7)
France (2017)	322	4.09	295	3.41	−16.6 (−18.0 to −15.2)	206	2.51	210	2.33	−7.2 (−7.9 to −6.5)
Germany	439	4.00	443	3.49	−12.8 (−13.6 to −12.0)	291	2.64	308	2.43	−8.0 (−8.6 to −7.4)
Greece	46	3.25	47	3.22	−0.9 (−1.1 to −0.7)	24	1.65	29	1.84	11.5 (8.7 to 14.3)
Hungary	84	6.51	74	5.54	−14.9 (−17.0 to −12.8)	56	3.76	46	3.07	−18.4 (−21.7 to −15.1)
Italy	311	4.05	367	4.18	3.2 (3.0 to 3.4)	195	2.46	221	2.42	−1.6 (−1.7 to −1.5)
Netherlands	160	6.97	133	5.29	−24.1 (−26.6 to −21.6)	103	4.60	99	4.02	−12.6 (−14.2 to −11.0)
Norway (2016)	60	9.63	48	6.92	−28.1 (−34.6 to −21.6)	35	5.71	32	4.76	−16.6 (−21.4 to −11.8)
Poland	272	5.72	254	4.76	−16.8 (−18.1 to −15.5)	188	3.54	157	2.83	−20.1 (−22.0 to −18.2)
Portugal	46	3.53	50	3.49	−1.1 (−1.3 to −0.9)	30	2.06	30	1.90	−7.8 (−9.8 to −5.8)
Romania	133	5.23	154	5.67	8.4 (7.5 to 9.3)	75	2.72	86	3.04	11.8 (10.1 to 13.5)
Serbia	75	7.44	74	7.07	−5.0 (−5.7 to −4.3)	41	3.86	35	3.23	−16.3 (−19.7 to −12.9)
Slovakia	41	6.24	51	6.59	5.6 (4.5 to 6.7)	28	3.83	28	3.58	−6.5 (−8.1 to −4.9)
Spain	169	3.15	175	2.66	−15.6 (−17.1 to −14.1)	109	1.98	115	1.70	−14.1 (−15.8 to −12.4)
Sweden	87	6.72	67	5.20	−22.6 (−26.1 to −19.1)	48	3.79	48	3.84	1.3 (1.1 to 1.5)
Switzerland	58	5.65	43	3.57	−36.8 (−43.3 to −30.3)	35	3.41	31	2.55	−25.2 (−30.7 to −19.7)
UK	385	4.98	399	4.67	−6.2 (−6.6 to −5.8)	269	3.38	254	2.89	−14.5 (−15.6 to −13.4)
EU	2608	4.64	2570	4.10	−11.6 (−11.9 to −11.3)	1669	2.85	1676	2.60	−8.8 (−9.1 to −8.5)
North America										
Canada	228	5.16	222	4.12	−20.2 (−21.9 to −18.5)	131	2.89	121	2.26	−21.8 (−24.2 to −19.4)
USA	2411	6.53	2082	4.71	−27.9 (−28.6 to −27.2)	1113	2.86	977	2.19	−23.4 (−24.3 to −22.5)
Latin America										
Argentina	138	3.83	142	3.42	−10.7 (−11.8 to −9.6)	78	1.99	76	1.65	−17.1 (−19.5 to −14.7)
Brazil	522	3.33	664	3.13	−6.0 (−6.3 to −5.7)	284	1.65	382	1.62	−1.8 (−1.9 to −1.7)
Colombia	80	2.44	109	2.35	−3.7 (−4.2 to −3.2)	61	1.60	81	1.53	−4.4 (−5.1 to −3.7)
Mexico	184	2.43	225	2.12	−12.8 (−13.9 to −11.7)	128	1.56	163	1.34	−14.1 (−15.6 to −12.6)
Venezuela (2016)	63	3.18	114	4.35	36.8 (30.5 to 43.1)	31	1.41	55	1.93	36.9 (27.8 to 46.0)
Australasia										
Australia	292	10.94	260	8.44	−22.9 (−24.7 to −21.1)	137	5.13	121	3.82	−25.5 (−28.5 to −22.5)
New Zealand	60	11.84	62	10.43	−11.9 (−14.4 to −9.4)	31	5.87	32	5.19	−11.6 (−15.0 to −8.2)
Japan	134	0.72	116	0.68	−5.6 (−6.2 to −5.0)	99	0.53	83	0.49	−7.5 (−8.5 to −6.5)
Philippines	60	1.07	96	1.15	7.5 (6.3 to 8.7)	34	0.56	53	0.60	7.1 (5.6 to 8.6)

ASMR, age-standardized (world population) mortality rate.

### Incidence

**Fig. 3:**
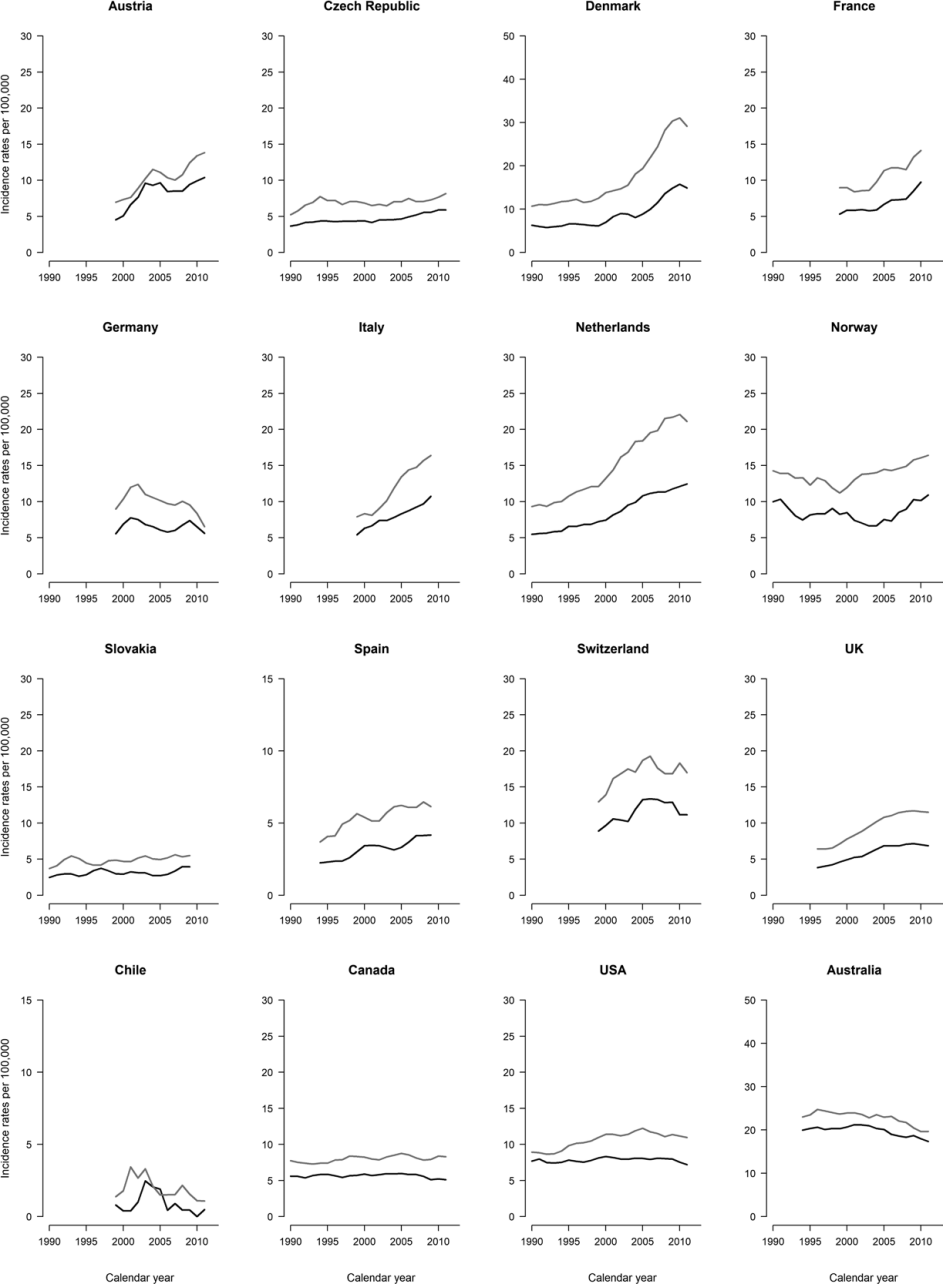
Annual age-standardized incidence rates from melanoma skin cancer per 100 000 in selected worldwide countries among men (black) and women (gray) aged 20–44.

**Fig. 4:**
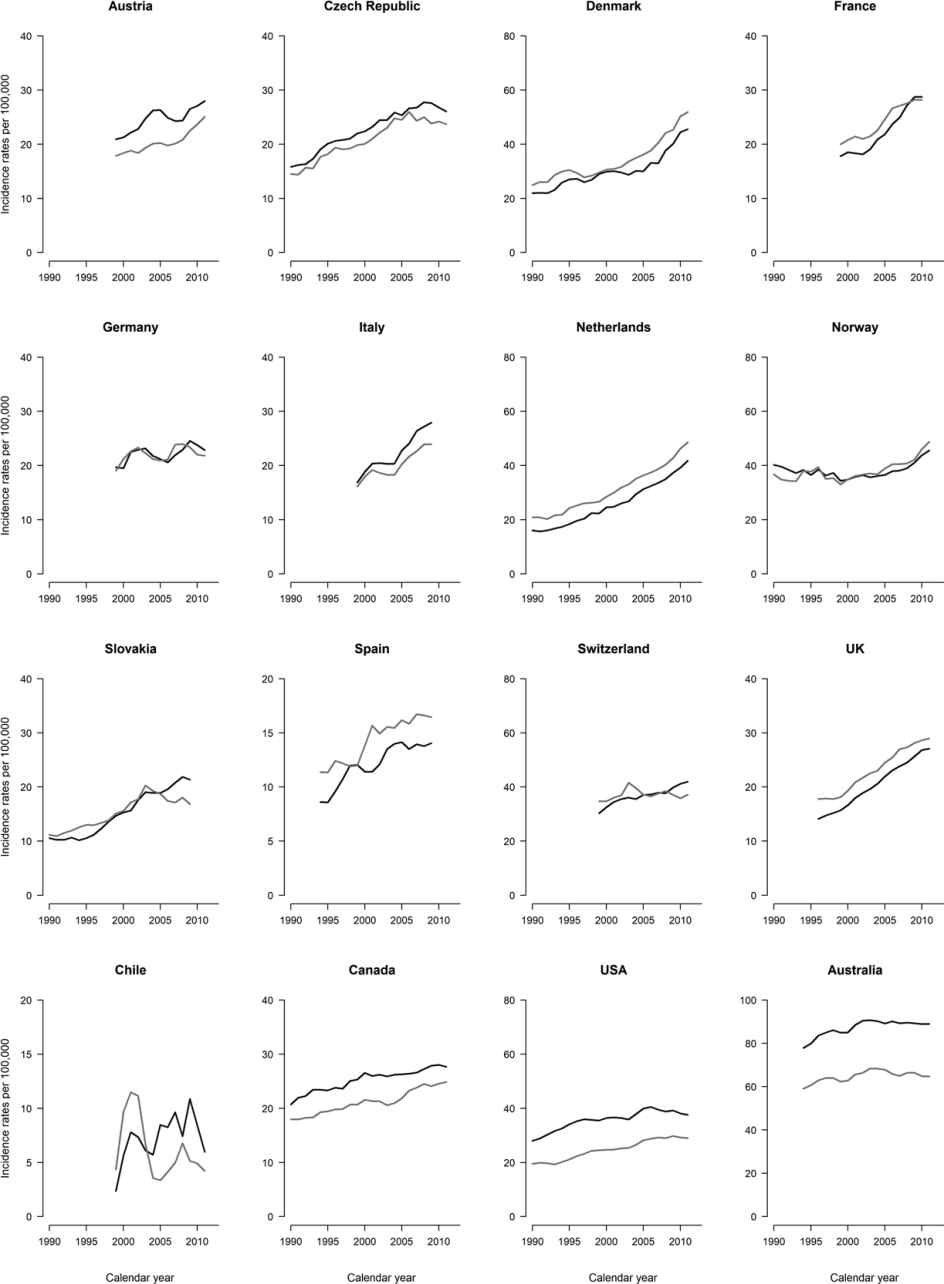
Annual age-standardized incidence rates from melanoma skin cancer per 100 000 in selected worldwide countries among men (black) and women (gray) aged 45–64.

Figure 3 illustrates the incidence trends for CMM among individuals aged 20–44 years in selected countries worldwide. Most countries showed increases in incidence patterns over time for both sexes, except for Canada, the USA, and Australia which reported stable trends as well as Israel, Japan, and the Philippines (data not shown). Middle-aged adults showed the same unfavorable trends over time in all countries considered except Australia (Fig. 4). Supplementary Table 2, Supplemental digital content 1, http://links.lww.com/MR/A372 provides the annual incidence average cases and age-standardized rates from CMM in 2008–2012 for both sexes and age groups. The highest rates and the largest average number of cases were recorded in Australia for both sexes and age groups. Supplementary Figures 1–2, Supplemental digital content 1, http://links.lww.com/MR/A372 illustrate the CMM incidence trends among adults aged 65 or older and at all ages, respectively. For both sexes, all countries showed steep increase among elderly and a smoother increment at all ages, with the highest rates recorded in Australia.

## Discussion

Mortality from CMM among young and middle-aged adults has shown a favorable pattern since the late 1980s in the majority of the European countries, the USA, Australia, and several Latin American countries. The greatest decline was observed in young adults aged 20–44 in Australia, New Zealand and in the USA, countries historically linked to the highest mortality [11–13]. In the European context, we observed a recent reduction in mortality rates in northern countries, such as Norway, Sweden, the Netherlands, and Denmark. This trend was also noticeable among middle-aged adults, albeit with a smaller proportional reduction. These findings align with previous research and further quantify reduction in mortality rates [3,4]. The continuing mortality decline in Australia and New Zealand in the last ten years (by 40 to 50%) confirms the success of the CMM prevention measure adoption in these countries.

Incidence showed upward trends in most European countries, whereas, in Australia, rates were stable for both sexes. The control of incidence trends in Australia is due to effective prevention programs, the control of UV exposure and sunburn, attention to early diagnosis and the widespread adoption of new melanoma detection technologies [14,15]. Another factor was an increased use of better-quality sunscreens.

Incidence trends may be influenced by a potential cohort effect. Young adults from countries with historically high rates exhibit stable or favorable trends, attributed to shifts in lifestyle, along with an increasing awareness of skin cancer and its prevention. The persistent rise in incidence among older adults also implies a cohort effect, stemming from generations characterized by low awareness and, consequently, prolonged exposure to UV.

The development of CMM has been linked to the accumulation of genetic mutations [12], triggered by exposure to UV radiation in fair-skinned populations, particularly during childhood, with a history of sunlight exposure and sunburns [12,16–21]. Indeed, countries with a light-skinned population, such as Australia, Western and Northern Europe, and North America, recorded high incidence rates. The higher incidence rates of countries such as southern Europe, Latin America, Australia, and New Zealand is also due to a geographical issue, that is, the greater proximity to the equator leading to a more frequent exposure to the sun. In contrast, the use of indoor tanning beds was common in northern Europe [22], the USA [23], and Australia [24], representing an additional risk factor in those populations for CMM.

The number, severity, and age of initial sunburn episodes can contribute to higher risk CMM. Sunburns tend to occur more frequently in fair skinned, red hair and blue-eyed individuals [12]. Additionally, subjects with high nevus count and the presence of dysplastic moles have an increased risk of developing CMM [12]. Other risk factors for CMM include a family or personal history of both CMM and other skin cancers, as well as immunosuppression [12].

Sunscreen plays a role in reducing CMM risk [25]. Cancer prevention authorities agree that reducing UV radiation exposure and promoting sun-protective behaviors are the best primary prevention. Many countries launched information campaigns to raise awareness about this practice (e.g. wearing UV protective clothing and avoid sun exposure between 11:00 a.m. and 3:00 p.m.) [26–28]. Southern hemisphere countries faced a higher burden of UV exposure due to ozone layer depletion. The most significant depletion of the ozone layer occurred in 1990 and 2000 [29]. By the early 2020s, ozone levels are expected to return almost to the same levels as in 1980, predicting a decrease in UV exposure.

Incidence and mortality trends also reflect the growing attention in prevention programs [12]. Better access to health care, early diagnosis, and the rise of effective therapies, even in advanced melanoma, explain the decrease in mortality rates [12]. Advances in biomolecular knowledge and therapeutic strategies, mainly immunotherapies, contributed to improved prognosis, even for those with advanced-stage CMM [12,30,31]. Shifts in demographic composition of a population must be considered as an influencing aspect of the incidence of CMM. High-income countries experienced a high migration inflow from low-income countries over 2000–2020. This phenomenon led to a change in the percentage of fair and dark-skinned population, the latter known to be less affected by CMM [32].

Women recorded higher CMM incidence rates but lower mortality rates. Differences in the quality of health care awareness, generally better for female patients worldwide, may play a role [33,34].

In interpreting the trends in skin cancer mortality, it is important to consider problems related to random variation, greater at younger ages and in smaller populations. To analyze incidence rates and trends, we retrieved data from CI5plus, which although often referring to subnational registries covering only a limited proportion of the national population, offers the most comprehensive data available for this purpose. Nonetheless, several countries with high data coverage and high data quality have been included in the present analysis and no inference was made on the statistical significance of rates and trends. Secondly, although the distinction between melanoma and non-melanoma skin cancer on death certificates is unreliable in most countries, this source of bias is restricted at the young age groups considered, where most deaths from skin cancer are due to melanoma. However, the distinction between melanoma and non-melanoma (squamous cells) skin cancer is considered valid for cancer registries included in the CI5 database considered in the present analysis.

Research and attention on melanoma have undergone a major surge since the 1980s. Particularly in 1985, ABCD (Asymetry, Border, Color and Diameter dermatological clinical criteria) was introduced as a tool for the patient and physicians for early diagnosis. From the 1990s onward, more and more clinical tools for early-stage melanomas (e.g. dermoscopy, confocal scanning laser microscopy) were developed. These two phenomena, along with increased education on prevention, had impacts on mortality and incidence between 2005 and 2009. In addition, new therapies for advanced melanomas were introduced after 2010, which led to further declines in mortality rates during 2015–2019 [15,30].

In conclusion, the incidence of CMM is still increasing in most countries providing valid data, while CMM mortality in young adults has been decreasing in the last three decades. This is related to increase in early CMM diagnosis together with primary prevention, control of UV exposure and sunburn, and improvement in management of the disease.

## Acknowledgements

This work was supported by AIRC Associazione Italiana per la Ricerca sul Cancro (project N. 22987) and CS and CLV were supported by EU funding within the NextGenerationEU-MUR PNRR (project N. PE00000007, INF-ACT). The funding sources had no role in the design and conduct of the study; collection, management, analysis, and interpretation of the data; preparation, review, or approval of the manuscript; and the decision to submit the manuscript for publication.

The data that support the findings of this study are openly available in WHO database at https://platform.who.int/mortality/themes/theme-details/topics/topic-details/MDB/malignant-neoplasms and in IARC’s Cancer Incidence in Five Continents (CI5) database at https://ci5.iarc.fr.

### Conflicts of interest

There are no conflicts of interest.

## Supplementary Material


