# Supplementary material for: Global trends in cutaneous malignant melanoma incidence and mortality
Source: Melanoma Res. 2024 Feb 21;34(3):265–75. doi: 10.1097/CMR.0000000000000959 (PMC11045545; doi:10.1097/CMR.0000000000000959)

SUPPLEMENTARY MATERIAL

Title: Global trends in cutaneous malignant melanoma incidence and mortality

Contents

Supplementary Table 1a. Results from joinpoint regression analysis for mortality from melanoma skin cancer, for men and women aged 20-44 years. 2

Supplementary Table 1b. Results from joinpoint regression analysis for mortality from melanoma skin cancer, for men and women aged 45-64 years. 3

Supplementary Table 2. Annual average incidence cases and age-standardized (world population) incidence rates from melanoma skin cancer per 100,000 person-years in selected countries worldwide in 2008-2012, according to sex and age groups. 4

Supplementary Figure 1. Annual age-standardized incidence rates from melanoma skin cancer per 100,000 in selected worldwide countries among men (blue) and women (red) aged 65+. 5

Supplementary Figure 2. Annual age-standardized incidence rates from melanoma skin cancer per 100,000 in selected worldwide countries among men (blue) and women (red) at all ages. 6

Supplementary Table 1a. Results from joinpoint regression analysis for mortality from melanoma skin cancer, for men and women aged 20-44 years.

	Men									Women								
**Country**	**Years1**	**APC1**	**Years2**	**APC2**	**Years3**	**APC3**	**Years4**	**APC4**	**AAPC**	**Years1**	**APC**	**Years2**	**APC2**	**Years3**	**APC3**	**Years4**	**APC4**	**AAPC**
France	1980-2017	-0.3							-0.3	1980-2012	-0.1	2012-2017	-6.8					-1
Germany	1980-2020	-2*							-2*	1980-1993	-0.7	1993-1997	-9.3	1997-2010	1.2	2010-2020	-5.7*	-2.3*
Italy	1980-2019	-0.5*							-0.5*	1980-2019	-0.4							-0.4
Poland	1980-1999	1.1	1999-2020	-2.8*					-1*	1980-1992	4*	1992-2020	-3.4*					-1.2*
Spain	1980-1989	8.4*	1989-2021	-2.6*					-0.3	1980-1994	4.7*	1994-2021	-2.3*					0
UK	1980-1990	1.1	1990-2015	-1.4*	2015-2020	-9.4*			-1.8*	1980-2020	-1.7*							-1.7*
EU-27	1980-1986	2.7*	1986-2010	-1.1*	2010-2018	-3.2*			-1*	1980-2005	-0.2	2005-2018	-2.9*					-1.1*
Argentina	1982-1997	3*	1997-2020	-0.8					0.7	1982-1987	-17.5*	1987-1999	8.9*	1999-2020	-1.8*			-0.8
Colombia	1984-2000	13*	2000-2020	-1.6					4.7*	1984-2000	6.5*	2000-2020	-0.5					2.6*
Canada	1980-2019	-2.4*							-2.4*	1980-2019	-2.4*							-2.4*
USA	1980-1985	7.2*	1985-1988	-10.3	1988-2020	-3.6*			-2.8*	1980-2013	-2.4*	2013-2020	-4.9*					-2.9*
Australia	1980-1994	1	1994-1997	-17.7	1997-2011	-1.1	2011-2021	-8.3*	-3.5*	1980-2011	-1.5*	2011-2021	-8.9*					-3.4*

APC: annual percent change. *Significantly different from 0 (p<0.05).

Supplementary Table 1b. Results from joinpoint regression analysis for mortality from melanoma skin cancer, for men and women aged 45-64 years.

	Men									Women								
**Country**	**Years1**	**APC1**	**Years2**	**APC2**	**Years3**	**APC3**	**Years4**	**APC4**	**AAPC**	**Years1**	**APC**	**Years2**	**APC2**	**Years3**	**APC3**	**Years4**	**APC4**	**AAPC**
France	1980-2009	1.1*	2009-2017	-3*					0.2	1980-1999	1.1*	1999-2017	-0.7					0.2
Germany	1980-1993	0.9*	1993-1996	-6.2	1996-2011	0.3	2011-2020	-2.9*	-0.7	1980-1994	0.1	1994-1997	-5.1	1997-2015	0.2	2015-2020	-4.8*	-0.9
Italy	1980-2019	-0.1							-0.1	1980-2019	0							0
Poland	1980-2003	1.5*	2003-2020	-2*					0	1980-1996	2.4*	1996-2015	-0.8*	2015-2020	-7.9*			-0.4
Spain	1980-1984	15.9*	1984-2021	-1*					0.5	1980-1996	3.9*	1996-2021	-0.9*					1*
UK	1980-1994	2.5*	1994-2016	0.2	2016-2020	-6.8*			0.3	1980-2009	-0.1	2009-2020	-2.3*					-0.7*
EU-27	1980-1988	2.3*	1988-2012	0.1	2012-2018	-3*			0.1	1980-1993	1.1*	1993-2013	0	2013-2018	-2.9*			0
Argentina	1982-1991	-4.2*	1991-1998	14.7*	1998-2020	-0.6			1.2	1982-2009	3.2*	2009-2020	-3*					1.4*
Colombia	1984-1993	-5.4	1993-1997	32.3	1997-2020	0.3			1.9	1984-1993	-8.1	1993-1999	21.9*	1999-2020	-0.4			0.9
Canada	1980-1996	1.6*	1996-2005	-2	2005-2009	5.5	2009-2019	-3.9*	-0.3	1980-2009	0	2009-2019	-2.8*					-0.7*
USA	1980-1987	3.9*	1987-2010	-1.1*	2010-2020	-4*			-1*	1980-1987	0.8	1987-2011	-1.2*	2011-2020	-3.3*			-1.3*
Australia	1980-2013	-0.5*	2013-2021	-6.7*					-1.7*	1980-2014	-0.7*	2014-2021	-6.5*					-1.7*

APC: annual percent change. *Significantly different from 0 ($p<0.05$).

Supplementary Table 2. Annual average incidence cases and age-standardized (world population) incidence rates from melanoma skin cancer per 100,000 person-years in selected countries worldwide in 2008-2012, according to sex and age groups.

	**Men**				**Women**			
	**Aged 20-44**		**Aged 45-64**		**Aged 20-44**		**Aged 45-64**	
	**Annual average incidence cases**	**Incidence rate**	**Annual average incidence cases**	**Incidence rate**	**Annual average incidence cases**	**Incidence rate**	**Annual average incidence cases**	**Incidence rate**
Austria	188	10.59	332	26.49	258	14.93	304	23.66
Czech Republic	139	6.42	400	26.92	187	9.37	352	23.64
Denmark	154	15.63	331	43.00	315	33.09	371	49.57
France	125	9.11	284	28.36	198	14.62	286	27.76
Germany	39	7.02	89	23.74	53	10.10	88	23.22
Italy	129	10.04	233	27.91	188	15.04	204	23.97
Netherlands	366	11.86	939	39.56	659	21.81	1057	45.68
Norway	97	9.84	282	43.00	152	16.92	284	45.61
Slovakia	51	4.60	146	21.37	67	6.14	129	16.84
Spain	94	4.69	157	14.04	127	6.67	184	16.44
Switzerland	57	11.43	138	40.93	87	18.12	128	37.34
UK	803	7.49	1948	26.19	1373	12.83	2148	28.52
Canada	268	5.53	1009	27.70	433	9.04	906	24.69
USA	438	8.19	1444	38.24	692	13.25	1147	29.20
Australia	820	19.60	2498	89.31	946	22.78	1843	65.76

Supplementary Figure 1. Annual age-standardized incidence rates from melanoma skin cancer per 100,000 in selected worldwide countries among men (blue) and women (red) aged 65+.

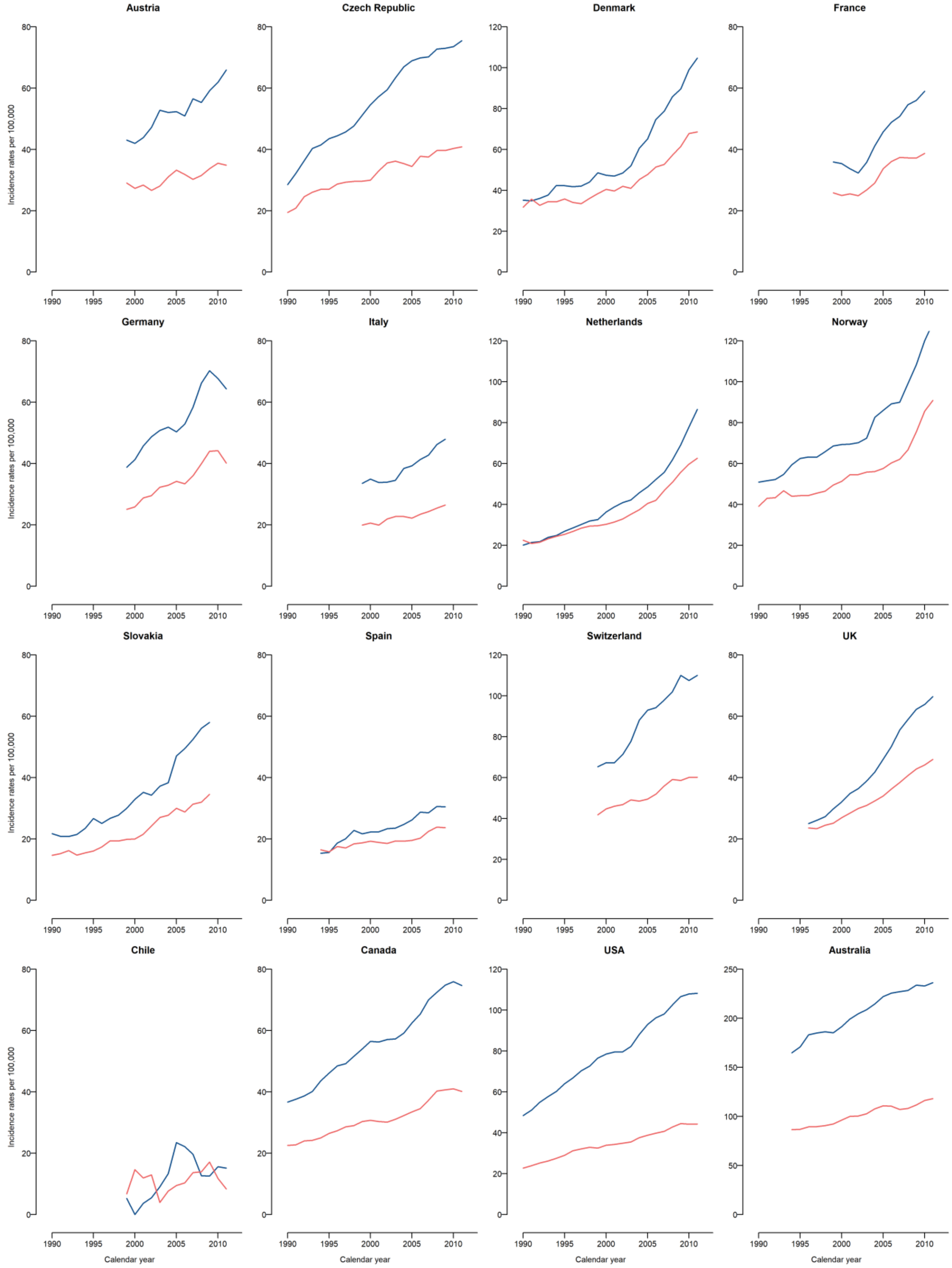

Supplementary Figure 2. Annual age-standardized incidence rates from melanoma skin cancer per 100,000 in selected worldwide countries among men (blue) and women (red) at all ages.

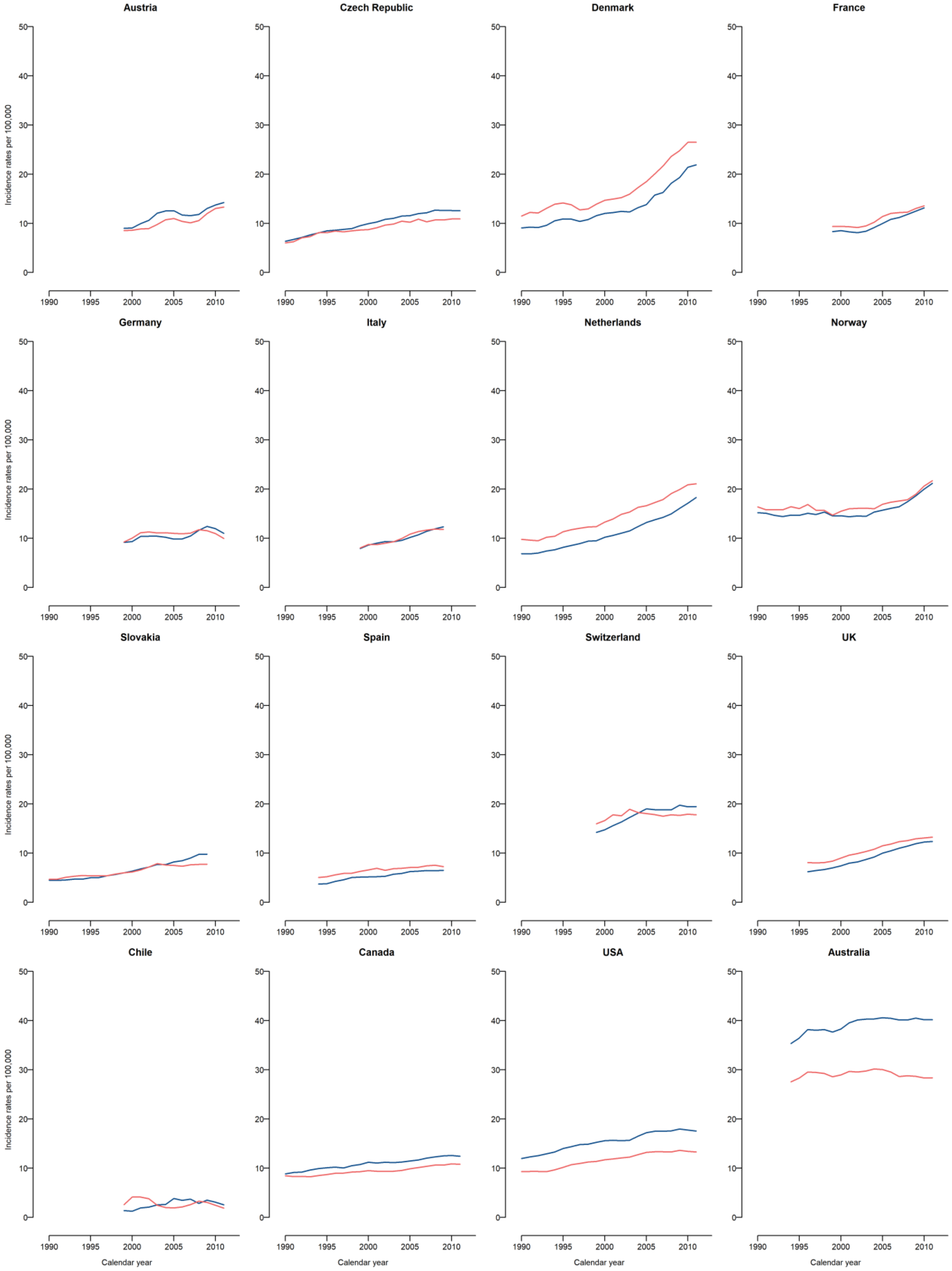

Supplement: Supplementary file 1 [file mr-34-265-s001.pdf]
